# VXX‐301, a muti‐epitope active immunotherapy targeting Tau, induces antibodies with favorable binding to Tau and exhibits efficacy *in vivo*

**DOI:** 10.1002/alz.089577

**Published:** 2025-01-09

**Authors:** Justin Boyd, JC Dodart, Stefan Prokop

**Affiliations:** ^1^ Vaxxinity, Merritt Island, FL USA; ^2^ University of Florida / Center for Translational Research in Neurodegenerative Disease, Gainesville, FL USA

## Abstract

**Background:**

Vaxxinity is developing an active immunotherapy targeting Tau for Alzheimer’s disease (AD) and other tauopathies. VXX‐301 is a multi‐epitope vaccine designed to target the N‐terminal and repeat domains of Tau. This design enables targeting multiple forms of Tau thought to contribute to Tau associated pathologies. Such a vaccine would be well positioned for the treatment and prevention of neuropathology induced by Tau.

**Method:**

VXX‐301 comprises a mixture of two Tau targeting peptides (p5555kb and p5187kb) each conjugated to Vaxxinity’s proprietary T helper carrier peptide and formulated with CpG and AdjuPhos. Antibody titers against specific targeted Tau regions in immunized rats were quantified and compared to single epitope targeting vaccines, p5555kb and p5187kb. Additionally, antibody titers against full length 2N4R rTau monomers and preformed fibrils were quantified. IgG isotyping was also conducted. The binding properties of VXX‐301‐derived antibodies were characterized by Western blot and immunohistochemistry. Epitope mapping was also conducted to confirm binding to targeted regions. *In vitro* activity was performed to quantify VXX‐301 antibodies in Tau aggregation and Tau uptake assays. *In vivo* efficacy was measured by evaluating the onset of ataxia and survival of P301L mice.

**Result:**

VXX‐301 induces high antibody titers against specific regions of Tau. Antibodies are primarily of the IgG1 isotype in rats and exhibit high binding potency to full length monomeric and PFF Tau. Additionally, antibodies bind to multiple recombinant Tau preparations, including 2N4R, 2N3R, 2N4R P301S, and K18 (P301L). Epitope mapping analyses confirm that VXX‐301‐derived antibodies bind to the intended epitopes of Tau. *In vitro*, VXX‐301‐derived antibodies block Tau cellular uptake as well as Tau aggregation. Finally, P301L transgenic mice immunized with VXX‐301 exhibit protection from ataxia onset and death.

**Conclusion:**

Here we present preclinical data indicating that VXX‐301 is immunogenic and induces antibodies with favorable characteristics. VXX‐301 antibodies exhibit binding to specific targeted regions as well as multiple forms of full‐length Tau. Moreover, we show that VXX‐301 antibodies bind to Tau in human brain tissue samples. VXX‐301 demonstrates both *in vitro* and *in vivo* efficacy against Tau‐dependent phenotypes, supporting the further development of this vaccine as a treatment and prevention of AD and other tauopathies.

